# PANoptosis-related genes in rheumatoid arthritis synovial tissue: screening, validation, and functional implications

**DOI:** 10.3389/fimmu.2026.1737366

**Published:** 2026-01-28

**Authors:** Yunmi Lu, Jiaxin Yang, Yasi Deng, Yuxin Chen, Ke Zhang, Liangcheng Ouyang, Yuge Zhai, Jinbo He, Chenxu Wang, Juan Huang, Wei Wang, Huanghe Yu

**Affiliations:** 1 TCM and Ethnomedicine Innovation & Development International Laboratory, School of Pharmacy, Hunan University of Chinese Medicine, Changsha, China; 2 Medical School, Hunan University of Chinese Medicine, Changsha, China

**Keywords:** bioinformatics, PANoptosis, RA synovial fibroblasts, rheumatoid arthritis, synovial hyperplasia

## Abstract

**Objective:**

To screen and validate the expression of key PANoptosis-related genes in the synovial tissue of rheumatoid arthritis (RA) through bioinformatics approaches and *in vitro*/*in vivo* experiments, explore their role in RA synovial hyperplasia, and provide new targets for RA treatment.

**Methods:**

Based on multiple genomic datasets from the GEO database, differentially expressed genes (DEGs) were screened using tools such as the limma package. The STRING database and Cytoscape 3.7.0 were employed to construct a Protein-Protein Interaction (PPI) network, followed by Gene Ontology (GO) functional annotation and Kyoto Encyclopedia of Genes and Genomes (KEGG) pathway enrichment analysis. Meanwhile, the CIBERSORT algorithm was used to perform immune infiltration analysis on the merged dataset to evaluate the proportional differences of 22 immune cell subsets. Classic *in vitro* and *in vivo* RA models (RA Fibroblast-Like Synoviocytes (RAFLSs) and adjuvant-induced arthritis (AIA) rat model) were established to investigate the expression of DEGs with high diagnostic value in RA synovial tissue.

**Results:**

Thirteen key DEGs involved in the PANoptosis pathway were identified in RA. GO and KEGG enrichment analyses showed that these genes were mainly involved in the NOD-like receptor signaling pathway and apoptosis signaling pathway. Immune infiltration analysis revealed significant differences in the immune microenvironment between RA patients and healthy controls: the RA group showed significant enrichment of pro-inflammatory immune cell subsets, while the control group was enriched with cells with inhibitory or homeostasis-maintaining functions. Compared with normal rats, the synovial tissue of AIA rats exhibited obvious abnormal hyperplasia. Western Blot (WB) results indicated that, compared with the control group, the expression levels of IL-18, NLRP3, GBP1, TNFSF10, Caspase-1, and Bcl-2 proteins in RAFLSs and AIA rat synovial tissue were significantly increased, while the expression levels of Bax and Caspase-3 were significantly decreased.

**Conclusion:**

During the pathogenesis of RA, the key PANoptosis markers (IL-18, NLRP3, GBP1, TNFSF10, Bax, Bcl-2, Caspase-1, and Caspase-3) are involved in invasive synovial hyperplasia. The characteristic immune cell imbalance in RA provides conditions for these key PANoptosis markers to regulate immune cell functions, and it is an important basis for the pathological changes of joint synovial hyperplasia in RA.

## 1 Introduction

Rheumatoid arthritis (RA) is a chronic, systemic autoimmune disease characterized by synovial hyperplasia in the joint cavity, pannus formation, and infiltration of various inflammatory cells (1). At present, the mechanism of its pathogenesis is not yet fully clear. RA Fibroblast-Like Synoviocytes (RAFLSs) are the main effector cells for inflammatory hyperplasia and invasion of joint synovium, and play an important role in the pathological development of RA (2); they can lead to the excessive secretion of chemokines, cytokines, and matrix-degrading molecules, thereby promoting immune cell infiltration and cartilage degradation (3). Therefore, RAFLSs can serve as key target cells for the proliferation of RA synovial tissue. Identifying targets that can regulate the proliferation of RAFLSs helps to clarify the pathogenesis of RA and provide new insights into the diagnosis and treatment of RA.

Apoptosis, pyroptosis, and necroptosis are well-studied programmed cell death modalities in genetics in recent years, and they play a crucial role in maintaining the balance between the body’s healthy state and disease conditions (4). PANoptosis is an integrated cell death program; it is not an independent pathway but is accomplished by coordinating these three key pathways—apoptosis, pyroptosis, and necroptosis—thereby highlighting the complex and subtle interactions and synergies among them. This program is typically activated when the body is confronted with severe infections or strong inflammatory stimuli (5, 6). Currently, PANoptosis is known to be closely associated with many diseases, as it can trigger excessive inflammation, cytokine storms, and tissue and organ damage (7). Studies have shown that the excessive proliferation and insufficient apoptosis of RAFLSs, as well as pyroptosis, play a key role in promoting RA-related damage and the persistent progression of inflammation (8). Furthermore, necroptosis may be involved in synovial angiogenesis in RA and is closely associated with the occurrence, development, and progressive exacerbation of RA (9, 10). The aforementioned studies suggest that apoptosis, pyroptosis, and necroptosis may occur simultaneously in RA patients, which indicates that PANoptosis may be closely involved in the pathological process of RA synovial hyperplasia and serves as an innovative target for RA mechanism research.

Against this backdrop, immune infiltration analysis could help to systematically reveal the composition, proportion, and functional status of immune cells in RA synovial tissue, clarify the role of different immune cell subsets in the formation and maintenance of the inflammatory microenvironment, and further identify key immune types associated with disease severity and progression (11). This analysis holds significant value for understanding the immunopathological mechanisms of RA, and is particularly helpful for elucidating the regulatory network between PANoptosis and immune cells: On one hand, PANoptosis may further recruit and activate immune cells by releasing inflammatory signals, exacerbating infiltration; On the other hand, immune cells infiltrated in synovial tissue may also regulate the occurrence of PANoptosis by secreting cytokines, forming a positive feedback loop that creates conditions for RA synovial hyperplasia.

**Figure 1:**
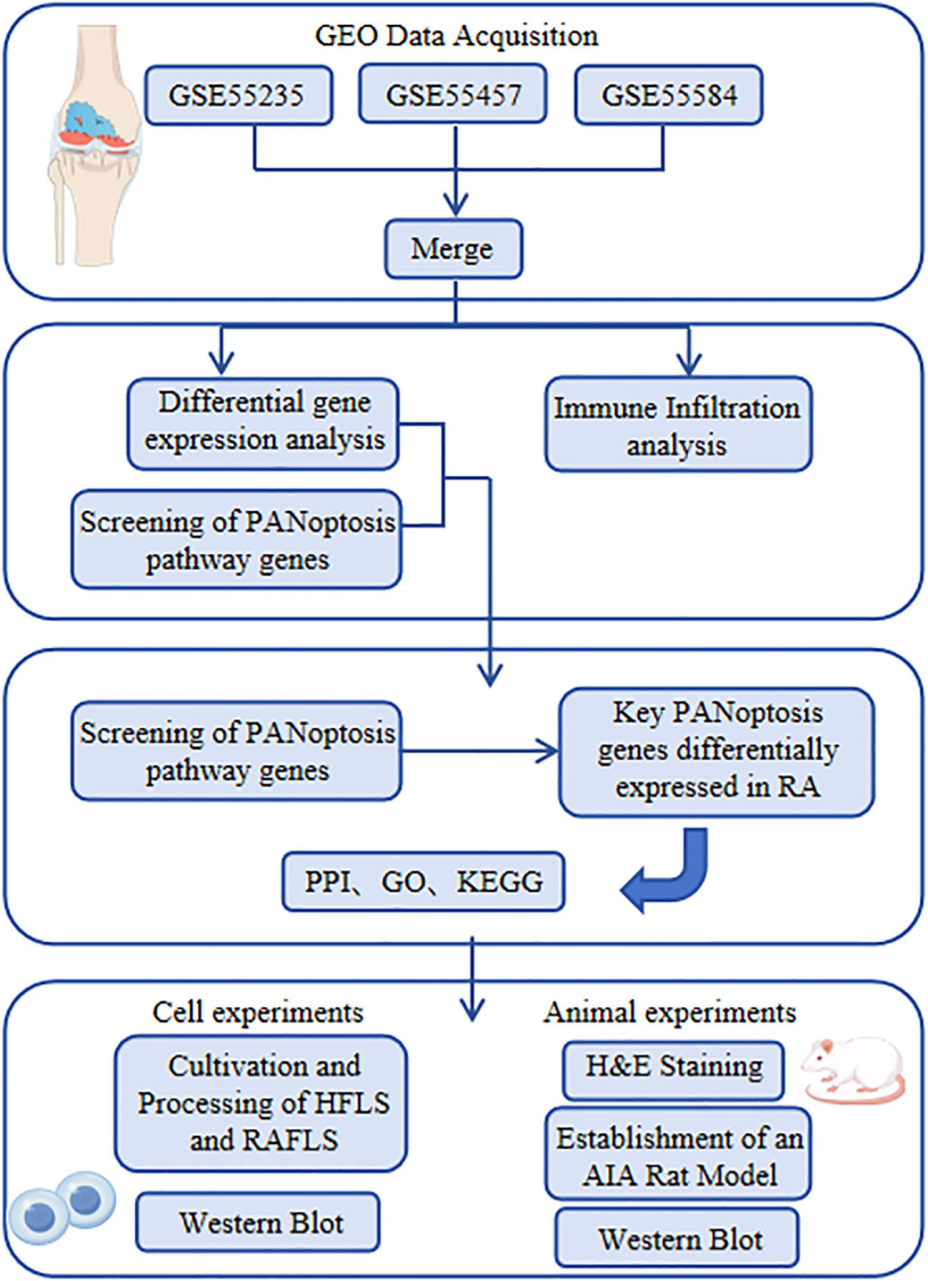
Research flowchart.

This study will use bioinformatics techniques and immune infiltration analysis techniques to identify potential PANoptosis-related genes and immune cells in human RA synovial tissue. Additionally, it will combine RAFLS and RA animal models to detect and validate the expression of these markers in RAFLSs and the synovial tissue of RA rats, thereby confirming the markers of PANoptosis during the pathogenesis of RA. This will provide new insights into the research on the pathogenesis of RA, facilitate the prediction of RA risk in subsequent studies, and support the early diagnosis and treatment of RA. The workflow of this study is shown in **Figure 1**.

## 2 Methods

### 2.1 Acquisition of human RA synovial tissue datasets

Gene expression data (GSE55235, GSE55457, GSE55584) were obtained from the NCBI Gene Expression Omnibus (GEO, https://www.ncbi.nlm.nih.gov/geo/). These datasets are publicly available, and their use in this bioinformatics analysis complies with the GEO data usage protocol. Human synovial tissue datasets—GSE55235, GSE55457, and GSE55584—were selected, which included 33 RA synovial tissue samples and 20 normal synovial tissue samples. The “hgu133a.db” and “dplyr” packages in R software were used to obtain probe IDs and gene symbols for each dataset. Multiple probes mapping to the same gene were aggregated by taking the maximum value to eliminate redundancy. Subsequently, the “limma” and “sva” packages in R software are utilized to read the three GEO datasets (GSE55235, GSE55457, GSE55584), convert them into matrices, and standardize the data to eliminate intra-array non-biological variation. Subsequently, we identified common genes across these three datasets. Although they all originated from the GPL96 platform with minimal platform variation, merging common genes further reduced platform-specific bias. These genes were consolidated into a new dataset. Group information for RA and normal samples across datasets was extracted. Batch correction was performed using the ComBat function in the “sva” package, applying both expression matrices and group information. The corrected expression data formed a new, complete dataset. To validate batch correction efficacy, principal component analysis (PCA) was conducted on the processed data. Visualization of PCA results assessed whether batch effects were successfully removed. Specifically, compare the sample clustering in PCA plots before and after correction. If samples from different original datasets no longer segregate by origin post-correction, batch effects are effectively controlled.

### 2.2 Analysis of differentially expressed genes in human RA synovial tissue

The “limma” and “stringr” packages in R software were invoked to analyze the merged dataset, so as to identify differentially expressed genes (DEGs) between the RA group and the normal group. Genes with a False Discovery Rate (FDR) < 0.05 and a gene expression difference of more than 1.5-fold (i.e., |log2 Fold Change (logFC)| > 0.585) were defined as DEGs. Compared with the normal group, among these DEGs, those with a positive logFC value were considered upregulated DEGs in the RA group, while those with a negative logFC value were regarded as downregulated DEGs in the RA group. The “ggplot2” and “pheatmap” packages in R software were used to generate volcano plots and heatmaps of RA-related DEGs, to visualize the expression results of these DEGs.

### 2.3 Immune infiltration analysis

The LM22 signature matrix, which provides gene expression signatures for 22 immune cell subtypes, was downloaded from the official CIBERSORT website. Based on this signature matrix, we performed immune cell infiltration analysis using the CIBERSORT algorithm on 33 RA synovial tissue samples and 20 normal synovial tissue samples in the merged dataset, with the help of packages including “e1071”, “preprocessCore”, “parallel”, and “bseqsc” in R software. First, the expression matrix was subjected to standardization and quantile normalization, and then the relative proportions of 22 immune cell subtypes in each sample were estimated. After obtaining the immune cell infiltration results, stacked bar charts were plotted to show the composition ratio of immune cells in each sample. Finally, samples were divided into the RA group and the Control group according to grouping information; the Wilcoxon rank-sum test was used to conduct inter-group difference analysis, and violin plots were used to visualize the immune cell subtypes with significant differences. The parameters applied in this study were as follows: (I) 100 deconvolutions (Perm) and (II) *P* < 0.05.

### 2.4 Acquisition of human PANoptosis pathway genes

**Figure 2:**
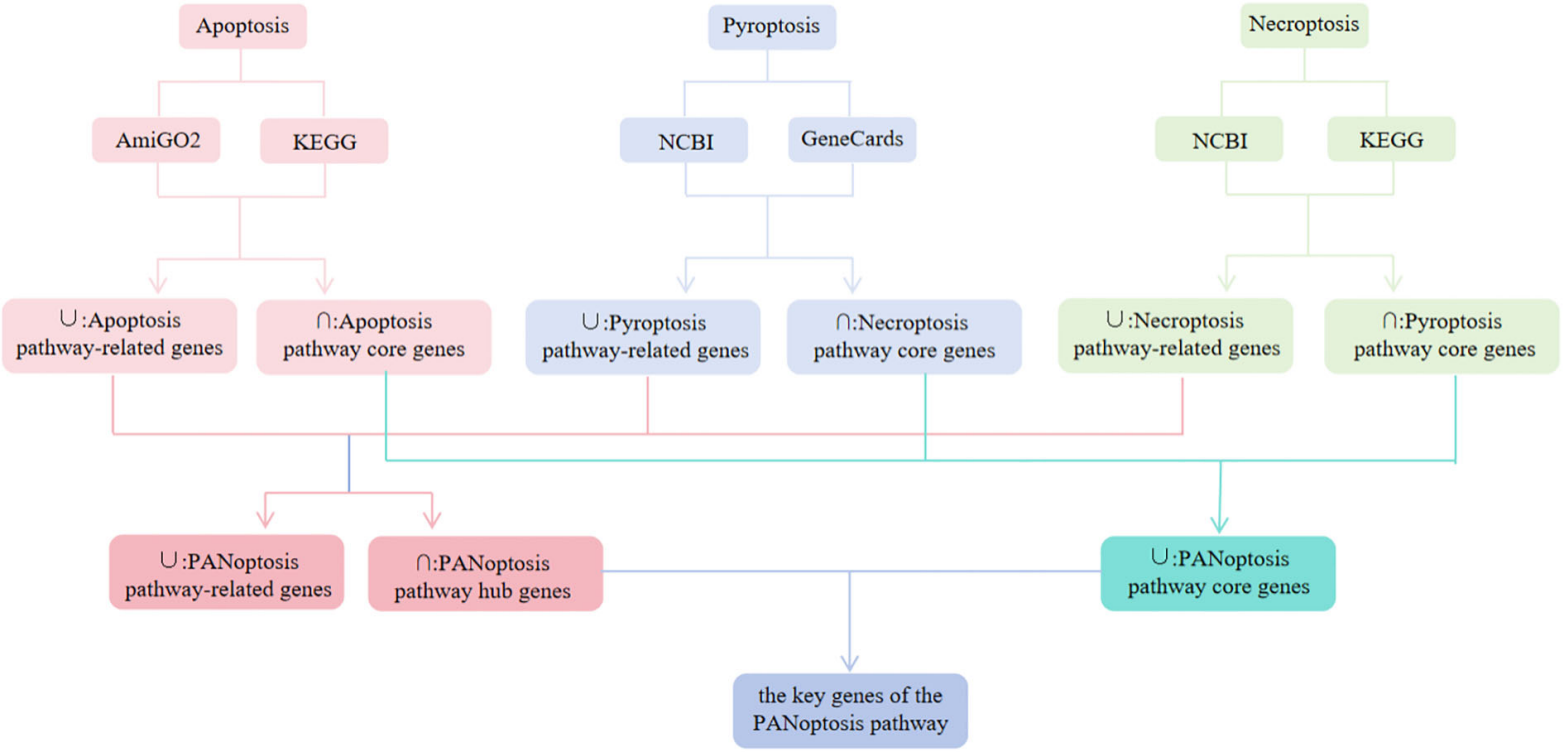
Identification of human PANoptosis pathway genes.

Apoptosis pathway genes were sourced from AmiGO2 (http://amigo.geneontology.org/amigo/landing) and the Kyoto Encyclopedia of Genes and Genomes (KEGG, https://www.kegg.jp/kegg/). Pyroptosis pathway genes were sourced from the National Center for Biotechnology Information (NCBI) and GeneCards (https://www.genecards.org), while necroptosis pathway genes were sourced from NCBI and KEGG. The construction of these gene lists relied on keyword searches: “apoptosis” was queried in AmiGO2 and KEGG, ‘pyroptosis’ in NCBI and GeneCards, and “necroptosis” in NCBI and KEGG. The species was set to “Homo sapiens,” the compartment selected as “pathway,” and the filtered genes were included. Genes from the same death pathway across different databases were unioned to form pathway-associated genes; their intersection constituted core genes. The combined sets of genes associated with apoptosis, pyroptosis, and necroptosis were collectively regarded as PANoptosis-related genes. Core genes collected from the apoptosis, pyroptosis, and necroptosis pathways were designated as PANoptosis core genes. The intersection of genes associated with these three pathways was identified as the PANoptosis hub genes. Both core and hub genes within the PANoptosis pathway are collectively termed PANoptosis pathway key genes, representing our primary focus (**Figure 2**).

### 2.5 Key differentially expressed PANoptosis genes in human RA

The differentially expressed genes derived from the human RA dataset were subjected to association analysis with the key genes of PANoptosis. The intersection of these two gene sets was defined as the key PANoptosis-related differentially expressed genes in RA. Subsequent analysis revealed the differential expression of these key PANoptosis genes across groups in the merged dataset.

### 2.6 Protein-protein interaction network analysis

The STRING database (https://cn.string-db.org/) was used to construct a Protein-Protein Interaction (PPI) network connecting the key PANoptosis-related differentially expressed genes in RA. Meanwhile, Cytoscape 3.7.0 was employed for the visualization of this PPI network, so as to obtain an interaction diagram between the PANoptosis genes.

### 2.7 GO and KEGG functional enrichment analysis

GO functional annotation and KEGG pathway enrichment analyses were performed on the key PANoptosis-related differentially expressed gene targets in human RA using the Metascape platform (https://metascape.org/). The species was set to “Homo sapiens”, Custom Analysis was selected, with the minimum count set to 3, P-value set to 0.01, and minimum enrichment factor set to 1.5. GO terms were visualized using the Bioinformatics Platform (https://www.bioinformatics.com.cn/).

### 2.8 Culture of human HFLS and RAFLS cells

#### 2.8.1 Materials

##### 2.8.1.1 Cells

HFLS (human fibroblast-like synoviocytes) cells were purchased from Beina Chuanglian Biotechnology Co., Ltd.; RAFLS (rheumatoid arthritis fibroblast-like synoviocytes) cells were obtained from the cell bank of the Chinese Academy of Sciences.

##### 2.8.1.2 Reagents

PBS (Lot No. WH0023Z271), 0.25% Trypsin Solution (Lot No. WHAB23D061), Fetal bovine serum (Lot No. SA210518), DMEM/F12 (Lot No. WHB823P251), and DMEM/High Glucose (Lot No. WHB823F311) were purchased from Wuhan Punoise Biotechnology Co., Ltd.; 1% Streptomycin/Penicillin (Lot No. 15070063) was purchased from Thermo Fisher Scientific, USA; Lipopolysaccharides (LPS, Catalog No. 00497693) were purchased from Sigma-Aldrich, USA; β-actin (Catalog No. AF7018), IL-18 (Catalog No. DF6252), NLRP3 (Catalog No. DF15549), GBP1 (Catalog No. DF3598), Bcl-2 (Catalog No. AF6139), Bax (Catalog No. AF0120), caspase-1 (Catalog No. AF5418), and caspase-3 (Catalog No. AF6311) antibodies were purchased from Jiangsu Qinke Biological Research Center Co., Ltd.; TNFSF10 (Catalog No. BS6907) antibody was purchased from Anolun (Beijing) Biotechnology Co., Ltd.; 5× Protein Loading Buffer (Batch No. 051823230907) was purchased from Shanghai Biyun Tian Biotechnology Co., Ltd.; Electrophoresis Buffer, Blotting Buffer, TBST, Rapid Blocking Solution, and 10-12.5% Colored Gel Preparation Kit were purchased from Beijing Saiwen Technology Co., Ltd.

##### 2.8.1.3 Instruments

Electrophoresis apparatus (DYCZ-25E) purchased from Beijing Liuyi Biotechnology Co., Ltd.; High-speed refrigerated centrifuge (ST8R) purchased from Thermo Fisher Scientific, Inc., USA; Cell counter (Countess3) purchased from Thermo Fisher Scientific, Inc., USA; Ultrasonic cell disruptor (900–92) purchased from Ningbo Xinzhi Biotechnology Co., Ltd.; Microplate reader (800 TS) purchased from Agilent Technologies, Inc., USA; Ultrapure water system (ZOOMAC-M) purchased from Hunan Zhongwo Water Environmental Technology Co., Ltd.

#### 2.8.2 Cell culture

The HFLS cell was cultured in high-glucose DMEM medium supplemented with 10% fetal bovine serum (FBS) and 1% penicillin-streptomycin double antibody solution; the RAFLS cell was cultured in DMEM/F-12 (1:1) medium supplemented with 10% fetal bovine serum (FBS) and 1% penicillin-streptomycin double antibody solution. Both cell lines were cultured in an incubator at 37 °C with 5% CO_2_ by volume. The cell status was observed regularly, and cells in the logarithmic growth phase were selected for the experiment.

### 2.9 Western blot detection of the expression of Key PANoptosis proteins in human HFLS and RAFLS cells

HFLS cells and RAFLS cells in the logarithmic growth phase were seeded into 6-well plates, respectively (RAFLS cells were induced into activated RAFLS with 500 ng/mL LPS). A mixed protein lysis buffer was prepared by mixing RIPA protein lysis buffer, PMSF, and phosphatase inhibitor at a ratio of 100:1:1. Cells were digested with trypsin, collected into 2 mL centrifuge tubes, washed 3 times with PBS, and 0.3 mL of protein lysis buffer was added to each tube. The cells were lysed on ice for 45 minutes to extract cellular proteins. After extracting proteins from HFLS cells and LPS (500 ng/mL)-induced activated RAFLS cells, the BCA kit was used for protein concentration quantification. 5× loading buffer was added, and the mixture was denatured at 95 °C for 10 minutes. 12.5% SDS-PAGE gels were prepared, with three biological replicates set for the normal group (HFLS) and three for the RA group (activated RAFLS). A 20 μg protein sample was loaded into each lane; electrophoresis was first performed at 80 V for 30 minutes, then at 120 V for 90 minutes. Subsequently, membrane transfer was conducted at 220 mA for 120 minutes. The membranes were incubated with primary antibodies (antibodies against IL-18/NLRP3/TNFSF10/GBP1/Bax/Bcl-2/Caspase-1/Caspase-3) at 4 °C overnight, followed by incubation with secondary antibody at room temperature for 2 hours, and then developed. The differential expression of target proteins between the two groups of cells was detected.

### 2.10 Establishment of an adjuvant arthritis rat model

#### 2.10.1 Animals

Fourteen male Sprague-Dawley (SD) rats, with a body weight of 70–90 g, were purchased from Beijing Weitong Lihua Experimental Animal Co., Ltd. The rats were housed in the SPF-grade Laboratory Animal Center of Hunan University of Chinese Medicine under the conditions of a room temperature of 20–24 °C, a relative humidity of 40%–50%, and a 12 h light/dark cycle. They were fed regularly and had free access to drinking water. All animal experiments were approved by the Ethics Committee of Hunan University of Chinese Medicine (Permit No. HNUCM21-2501-20).

#### 2.10.2 Establishment of the AIA model and sample collection

The 14 SD rats were randomly divided into a normal group and a model group, with 7 rats in each group. For the model group, 0.2 mL of Complete Freund’s Adjuvant (CFA) containing 400 μg of heat-inactivated Mycobacterium tuberculosis (Mtb) was subcutaneously injected at approximately 3 cm from the root of the rat’s tail; the normal group was injected with an equal volume of normal saline as a control. On the 14th day after modeling, obvious redness and swelling of the paws were observed in the model group, indicating the successful establishment of the arthritis model. At the experimental endpoint, Anesthesia was induced by placing each rat in a sealed induction chamber connected to a calibrated vaporizer. A mixture of oxygen and isoflurane was delivered at a flow rate of 600 mL/min. The isoflurane concentration was set at 3% (v/v) for induction. After approximately 3 minutes, loss of righting reflex confirmed the onset of anesthesia. The deeply anesthetized rat was then quickly transferred to a surgical platform in a supine position. Anesthesia was maintained via a nose cone with an isoflurane concentration reduced to and maintained at 2.5% (v/v). The depth of surgical anesthesia was verified by the absence of a pedal withdrawal reflex. Subsequently, terminal blood collection was performed via the abdominal aorta using a sterile syringe. Exsanguination under deep anesthesia served as the method of euthanasia, with death confirmed by the cessation of spontaneous breathing and the absence of a palpable heartbeat. Following confirmation of death, the right hind paw was promptly severed using sterile bone scissors. The synovial tissue was carefully dissected, immediately snap-frozen in liquid nitrogen, and stored at -80 °C for subsequent protein extraction.

### 2.11 Western blot detection of the expression of key PANoptosis proteins in synovial tissue of normal and AIA rats

Synovial tissue samples from the joints of rats in the normal control group and the AIA model group were cut into small pieces, with each sample weighing approximately 30 mg. The tissue pieces were then added to RIPA lysis buffer (supplemented with PMSF and phosphatase inhibitor) and homogenized via ultrasonic homogenization. The homogenized mixture was centrifuged (4 °C, 12,000 rpm, 15 min), and the supernatants containing total protein were collected. The total protein concentration in the supernatants was quantified with a BCA protein assay kit. Total protein samples were mixed with 5× loading buffer and denatured by heating at 95 °C for 10 minutes, followed by sodium dodecyl sulfate-polyacrylamide gel electrophoresis (SDS-PAGE) for protein separation. The separated proteins were transferred onto a polyvinylidene fluoride (PVDF) membrane. The membrane was then blocked with 5% non-fat milk in TBST buffer at room temperature for 1 hour, followed by incubation with primary antibodies (against IL-18, NLRP3, GBP1, TNFSF10, Caspase-1, Caspase-3, Bax, and Bcl-2) at 4 °C overnight. Subsequently, the membrane was incubated with horseradish peroxidase (HRP)-conjugated secondary antibodies at room temperature for 2 hours. Protein bands were visualized using an enhanced chemiluminescent (ECL) detection system. The grayscale values of the protein bands were quantified using ImageJ software, which were used to represent the expression levels of the target proteins. Finally, the expression levels of these key PANoptosis-related proteins were compared between the normal control group and the AIA model group.

### 2.12 Histopathological analysis

The excised left hind paw of the rat was fixed in 4% paraformaldehyde for 1 week. Subsequently, decalcification was performed using 10% EDTA, which lasted for 2 months. After decalcification, the paws were embedded in molten paraffin; the paraffin was then cooled and solidified before the paws were cut into thin sections. The sections were stained with hematoxylin and eosin (H&E), and the tissue morphology was observed under a light microscope.

### 2.13 Data analysis

Experimental data are expressed as mean ± standard deviation (mean ± SD). Image analysis was performed using ImageJ, while graphing and statistical analysis were conducted with GraphPad Prism 9.0. For comparisons between two groups, an independent-samples t-test was used; for comparisons among multiple groups, one-way analysis of variance (ANOVA) was employed. Statistical significance was defined as *P <* 0.05.

## 3 Results

### 3.1 Analysis of differentially expressed genes in human RA synovial tissue

**Figure 3:**
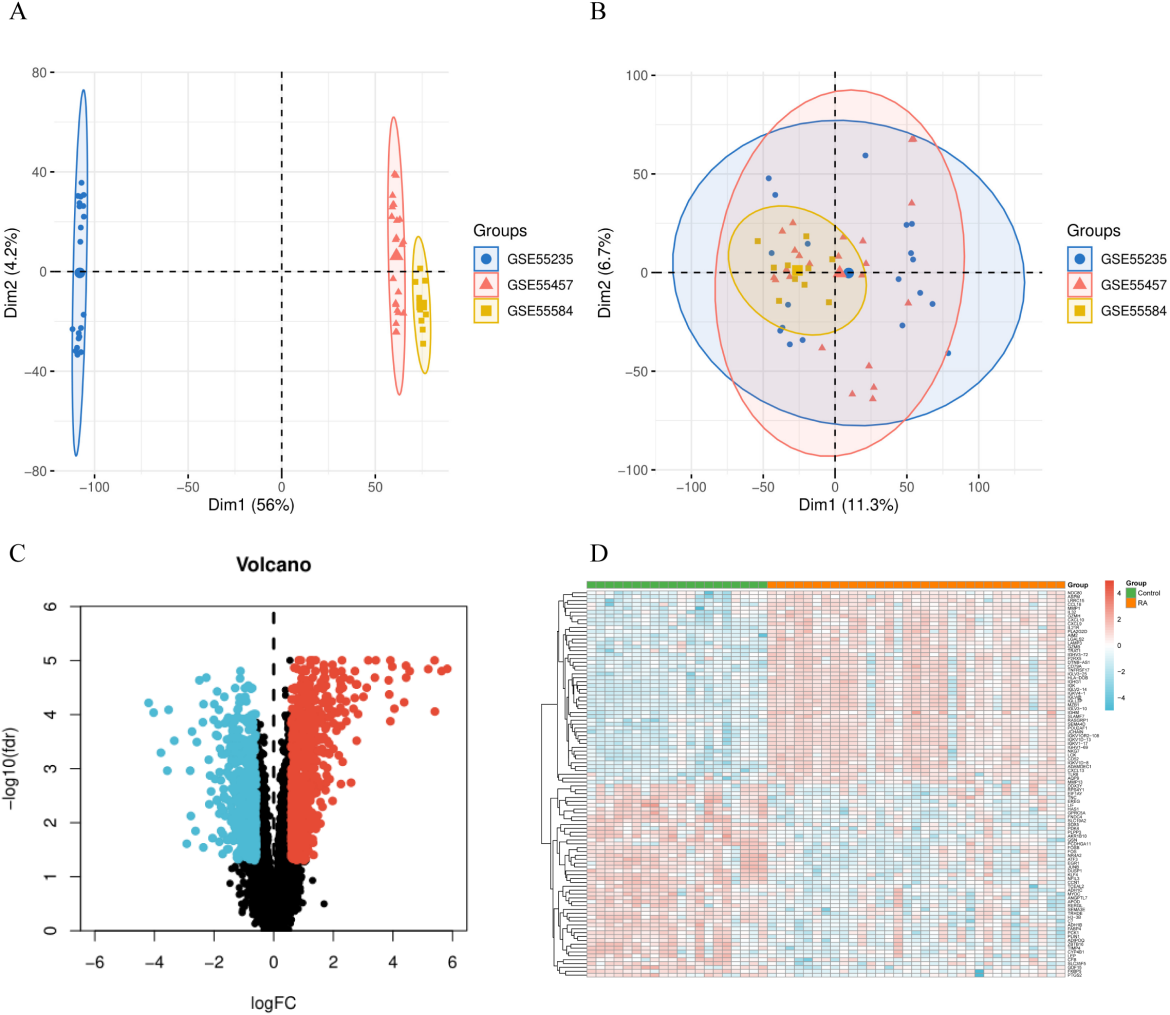
RA differentially expressed genes. **(A, B)** Principal Component Analysis (PCA) of gene expression levels before and after batch processing. **(C)** Volcano plot of RA differentially expressed genes. **(D)** Heatmap of RA differentially expressed genes.

GSE55235, GSE55457, and GSE55584 datasets were merged and subjected to batch correction after being processed (**Figure 3A, B**). Heatmaps and volcano plots illustrating differentially expressed genes in RA were generated using the “pheatmap” and “ggplot2” packages in R, based on 33 RA synovial tissue samples and 20 normal synovial tissue samples. Subsequent analysis identified 1,293 differentially expressed genes (with the cutoff criteria of FDR < 0.05 and |logFC| > 0.585). The volcano plot (**Figure 3C**) displays the logFC values and statistical significance of all genes, where points closer to the plot edges (indicating high |logFC|) and higher up (indicating low FDR) represent greater statistical significance. Red dots represent differentially expressed genes that are upregulated in RA patients relative to normal samples, blue dots represent those that are downregulated, and black dots represent genes with no significant differences in expression.

A heatmap (**Figure 3D**) was constructed using the top 100 most significantly differentially expressed genes, including the top 50 upregulated and bottom 50 downregulated ones. Columns in the heatmap correspond to the 53 samples (20 normal samples on the left and 33 RA samples on the right), while rows represent the 100 differentially expressed genes. The heatmap reveals that apoptosis-related genes (e.g., GZMB, GZMH), pyroptosis-related genes (e.g., AIM2, TLR8), and necroptosis-related genes (e.g., TNFRSF17) are all significantly upregulated in RA samples. This finding indicates that in RA synovial tissue, activation is not limited to a single cell death pathway; instead, a PANoptosis network involving multiple synergistic pathways exists. These differentially expressed genes may serve as the core molecular nodes of this PANoptosis network. We hypothesize that this PANoptosis state in RA synovial tissue is closely associated with RA pathogenesis and the progression of synovial hyperplasia, thereby contributing to pathological damage in RA.

### 3.2 Immune infiltration analysis

**Figure 4:**
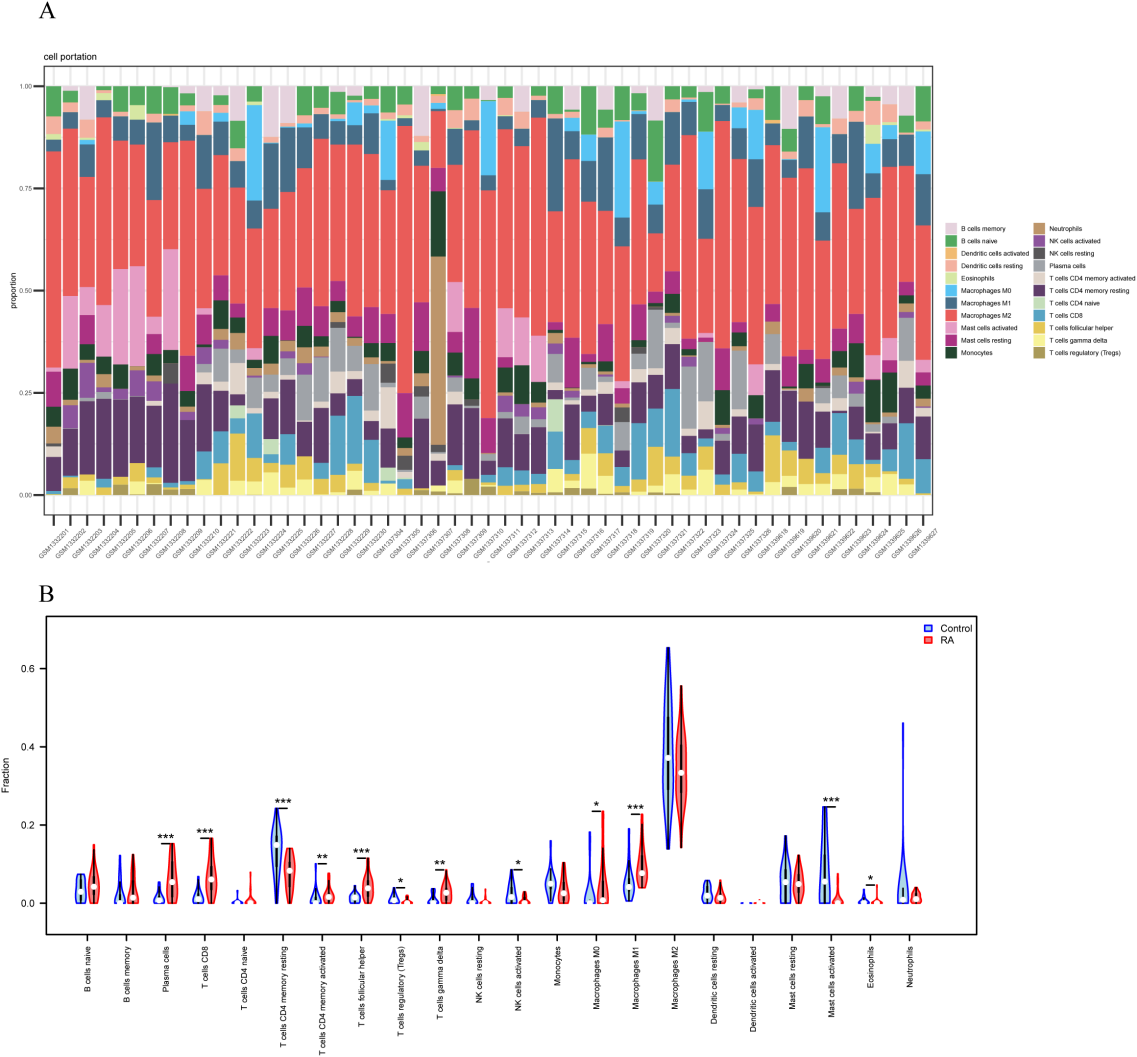
Immunological infiltration analysis. **(A)** Relative proportions of 22 immune cell subpopulations in 53 samples calculated using the CIBERSORT algorithm. **(B)** Differences in 22 immune cell subpopulations between normal and RA synovial tissues (blue represents the normal group, red represents the RA group).

Immune infiltration analysis of the experimental dataset using CIBERSORT revealed significant differences in the proportions of 22 immune cell types across each sample. As shown in **Figure 4A**, the bar charts clearly illustrate the immune cell composition of each sample; **Figure 4B**’s violin plot further reveals that the RA group exhibits significantly higher proportions of M0 macrophages, M1 macrophages, plasma cells, CD8^+^ T cells, and helper T cells compared to the control group. Conversely, the control group shows relatively higher proportions of regulatory T cells, activated mast cells, activated NK cells, and resting memory CD4^+^ T cells. These findings reveal marked differences in the immune microenvironment between RA patients and healthy controls: the RA group predominantly harbors pro-inflammatory or effector immune cell subsets, whereas the control group is characterized by cells with immunosuppressive or homeostasis-maintaining functions.

Abnormal changes in synovial tissue are a typical pathological feature of RA, primarily driven by the excessive proliferation of FLS and the extensive infiltration of immune cells such as T cells, B cells, and macrophages (12). Within the synovial tissue, these infiltrating immune cells not only directly induce synovial inflammation by secreting multiple pro-inflammatory factors but also form complex interactive networks with synovial cells, jointly driving synovial hyperplasia and joint damage (13). Notably, FLS are not passive bystanders in RA progression. Instead, they undergo abnormal activation and directly participate in synovial hyperplasia. These cells engage in strict bidirectional crosstalk with infiltrating immune cells, cytokines, and proteases, further amplifying the inflammatory and tissue destruction processes (14).

In this microenvironment, infiltrating immune cells, particularly M1 macrophages and CD8^+^ T cells, can directly induce PANoptosis by releasing proinflammatory factors such as TNF-α, IFN-γ, and IL-1β, as well as cytotoxic molecules like granzyme and perforin (15, 16). Conversely, PANoptosis further exacerbates immune infiltration: dying cells release substantial quantities of damage-associated molecular pattern (DAMP) molecules, including ATP, HMGB1, and DNA fragments. These molecules act as chemotactic signals, recruiting additional immune cells to the inflammatory site, intensifying the local inflammatory response, and forming a self-amplifying positive feedback loop. This mechanism plays a significant role in severe conditions such as sepsis and cytokine storms, where it leads to the massive release of factors like IL-1β and IL-18, triggering systemic hyperinflammation and multiple organ failure (15, 16).

Therefore, the characteristic immune cell infiltration pattern revealed in RA in this study not only explains the immunological basis of synovial hyperplasia and chronic inflammation at the cellular level but also suggests that PANoptosis is likely the key bridge connecting abnormal immune activation to joint tissue damage. Specifically, infiltrating pro-inflammatory immune cells drive abnormal activation of FLS and synovial hyperplasia through direct contact and secretion of multiple effector molecules, while simultaneously inducing PANoptosis. Mediators released during PANoptosis, such as DAMPs, further recruit and activate immune cells, forming a positive feedback loop that continuously exacerbates synovial pathology. In this process, key PANoptosis markers may play a crucial role in regulating the functional activity and differentiation status of dysregulated immune cells. This study provides a theoretical basis and candidate research directions for subsequent screening and validation of PANoptosis markers highly correlated with RA pathology.

### 3.3 Analysis of key genes in the human PANoptosis pathway

**Figure 5:**
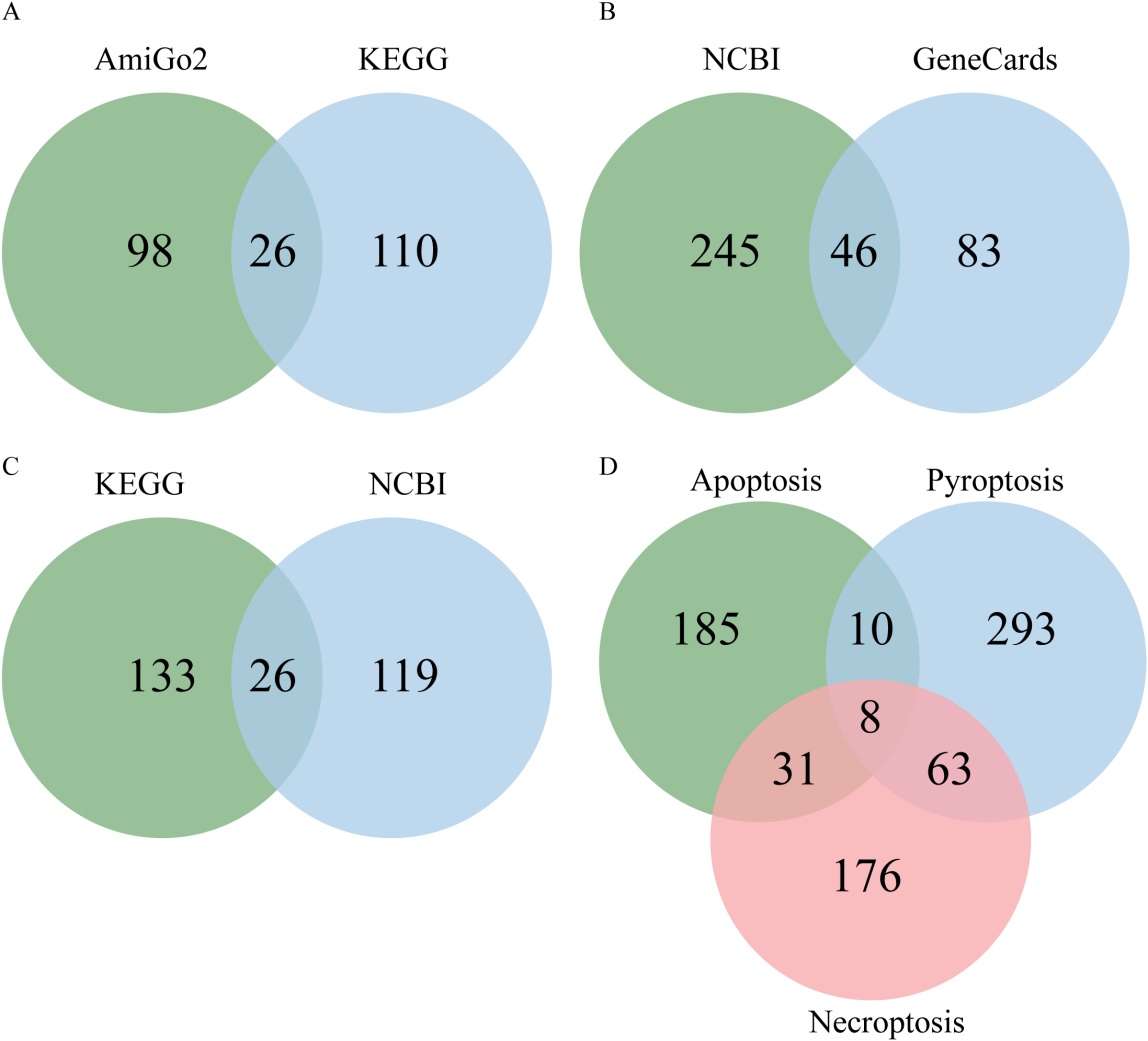
Acquisition of PANoptosis pathway genes. **(A)** Apoptosis pathway genes; **(B)** Pyroptosis pathway genes; **(C)** Necroptosis pathway genes; **(D)** PANoptosis pathway hub genes. The two different colors represent the data sources. The apoptosis pathway genes were obtained from two different databases or resources. The pyroptosis pathway genes were obtained from two different databases or resources. The necroptosis pathway genes were obtained from two different databases or resources. The PANoptosis pathway hub genes were identified as the intersection of the genes from the three cell death pathways (apoptosis, pyroptosis, and necroptosis). This figure illustrates the process of obtaining the genes related to the three cell death pathways and the subsequent identification of the core PANoptosis pathway genes and hub genes through data integration and analysis.

A total of 124 and 136 apoptosis pathway genes were obtained from the AmiGo2 and KEGG databases, respectively. The union of these two datasets yielded 234 apoptosis pathway-related genes, while their intersection identified 26 core apoptosis pathway genes (**Figure 5A**). For the pyroptosis pathway genes, 291 and 129 genes were retrieved from the NCBI and GeneCards databases, respectively. The union of these two gene sets resulted in 374 pyroptosis-related genes, and their intersection defined 46 core pyroptosis pathway genes (**Figure 5B**). Regarding necroptosis pathway genes, 159 and 145 genes were obtained from the KEGG and NCBI databases, respectively. Their union produced 278 necroptosis-related genes, and their intersection identified 26 core necroptosis pathway genes (**Figure 5C**; **Table 1**). The core genes of the three cell death pathways—apoptosis, pyroptosis, and necroptosis—together constitute the core genes of the PANoptosis pathway, totaling 83 genes. The union of genes associated with these three pathways yielded 766 PANoptosis-related genes, while their intersection identified 8 PANoptosis pathway hub genes (**Figure 5D**): TNFSF10, TNF, CASP8, BAX, CAPN1, TP53, NFKB1, and PYCARD. Merging the core genes and hub genes of the PANoptosis pathway resulted in the key genes of the PANoptosis pathway, which totaled 85 genes.

**Table 1:** Acquisition of PANoptosis pathway-related genes.

Geen name	Apoptosis	Pyroptosis	Necroptosis
AmiGo2	124	-	-
KEGG	136	-	159
NCBI	-	291	145
GeneCards	-	129	-
Union-Related Genes	234	374	278
Intersection-Core Genes	26	46	26

### 3.4 Key PANoptosis pathway genes differentially expressed in human RA disease

**Figure 6:**
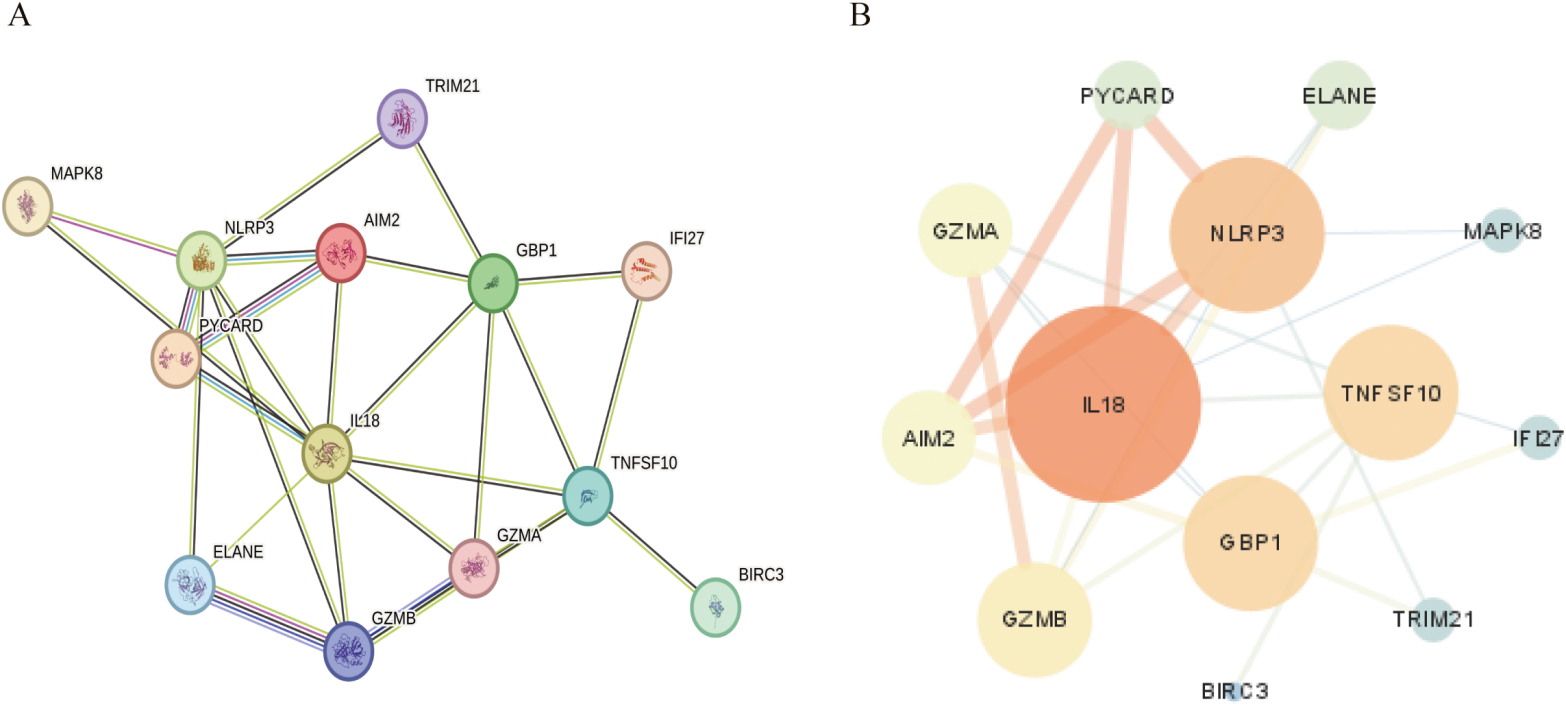
Protein-protein interaction network of key PANoptosis differentially expressed genes in RA disease. **(A)** Protein-protein interaction network diagram. **(B)** Cytoscape visualization of the PPI network, sorted by degree value distribution.

The intersection of the key genes of the PANoptosis pathway and the differentially expressed genes in human RA synovial tissue yielded 13 genes, referred to as the key PANoptosis pathway genes in RA. These genes are AIM2, ELANE, GBP1, GZMA, GZMB, IFI27, IL18, NLRP3, PYCARD, TRIM21, BIRC3, MAPK8, and TNFSF10. PPI network analysis (**Figure 6**) revealed the differential expression of these 13 key genes in the RA disease dataset and their protein interaction network relationships.

**Figure 7:**
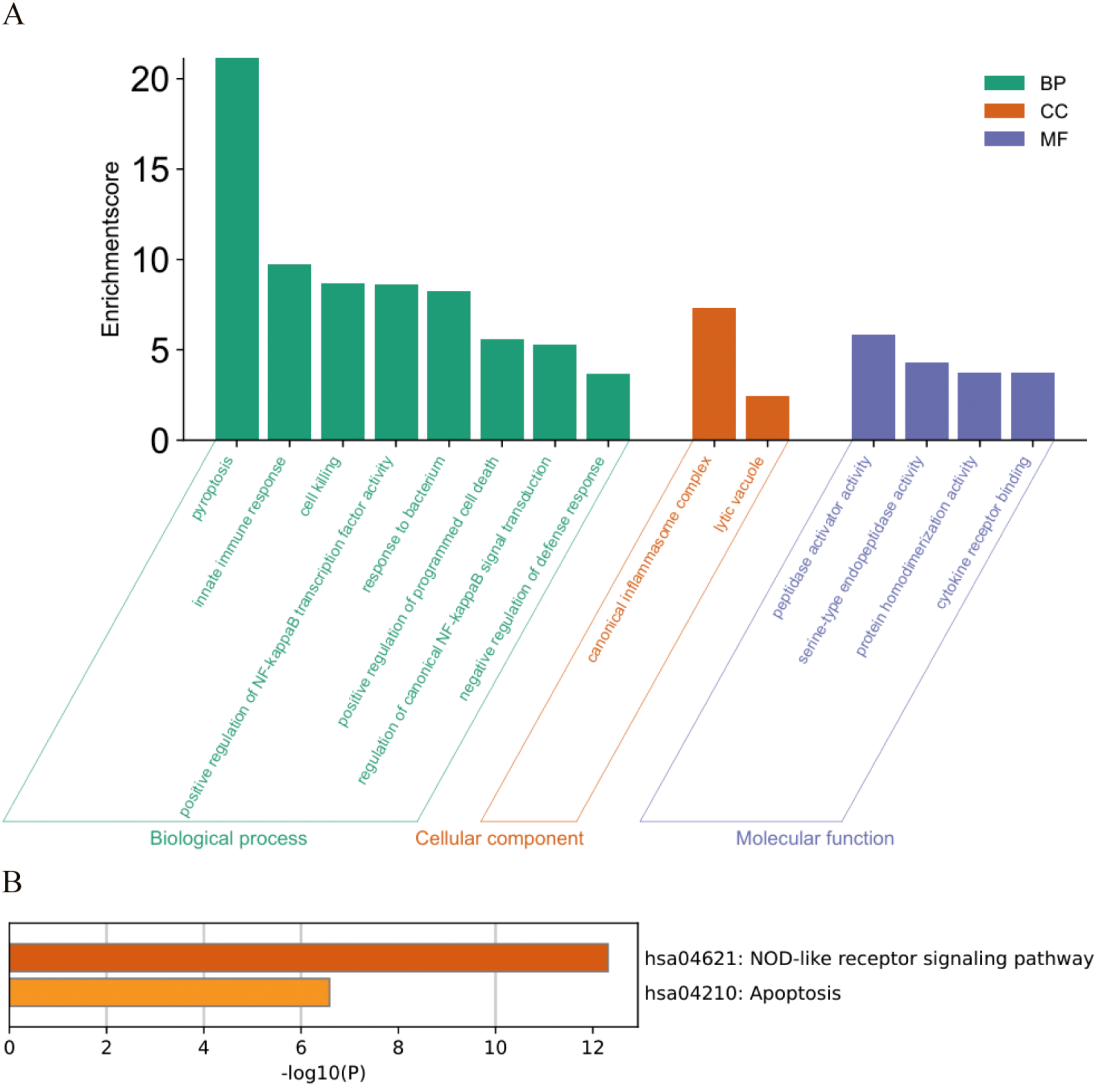
GO and KEGG functional enrichment of key PANoptosis differentially expressed genes in RA disease. **(A)** GO functional enrichment results. **(B)** KEGG functional enrichment results.

GO functional enrichment analysis and KEGG pathway enrichment analysis were performed on the key PANoptosis-related differentially expressed genes in human RA. The results of GO and KEGG enrichment analyses showed that these differentially expressed genes are mainly associated with biological processes such as pyroptosis, innate immune response, and cell death; cellular components including inflammasome and lytic vacuole; and molecular functions like peptidase activator activity and serine-type endopeptidase activity (**Figure 7A**). Additionally, these genes are widely involved in signaling pathways such as the NOD-like receptor signaling pathway and the apoptosis signaling pathway (**Figure 7B**).

### 3.5 Differences in expression levels of key PANoptosis proteins in HFLS and RAFLS cells

**Figure 8:**
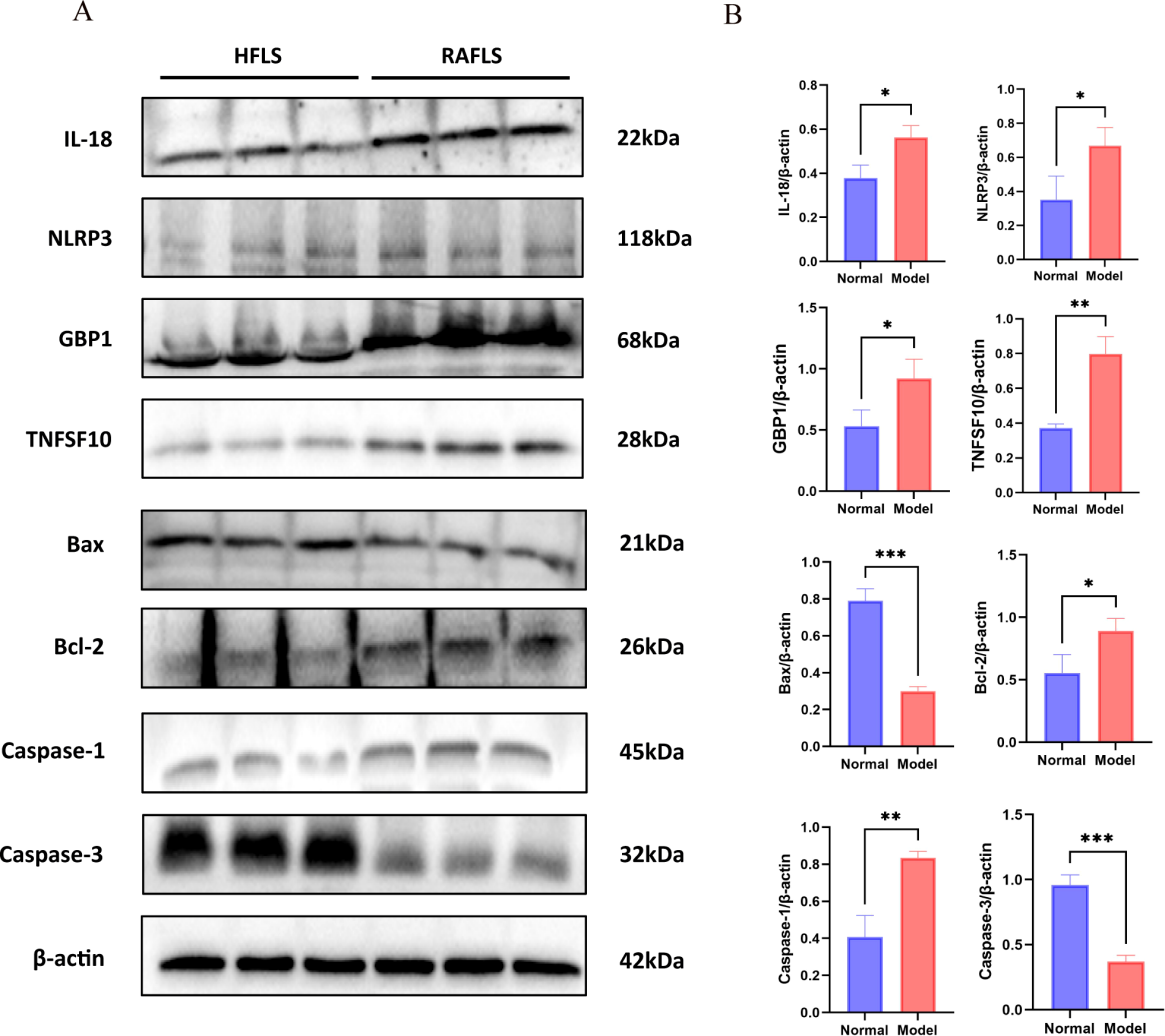
Differences in expression levels of key PANoptosis proteins in HFLS and RAFLS cells. Compared with the HFLS group, *P < 0.05, **P < 0.01, ***P < 0.001. **(A)** Western blot results. **(B)** Differential analysis between the normal group and the RA group.

As shown in **Figure 8**, compared with HFLS cells, the expression levels of key PANoptosis proteins in RAFLS cells—including IL-18 (*P* < 0.05), NLRP3 (*P* < 0.05), GBP1 (*P* < 0.05), TNFSF10 (*P* < 0.01), Caspase-1 (*P* < 0.01), and Bcl-2 (*P* < 0.05)—were significantly increased, while the expression levels of Caspase-3(*P* < 0.001) and Bax (*P* < 0.001) were significantly decreased. The results demonstrated that the expression of PANoptosis-related proteins was significantly elevated in RAFLS cells, indicating that RA synovial hyperplasia is closely related to the occurrence of PANoptosis in RAFLS cells.

### 3.6 Paw swelling and pathological changes in normal rats and adjuvant-induced arthritis rats

**Figure 9:**
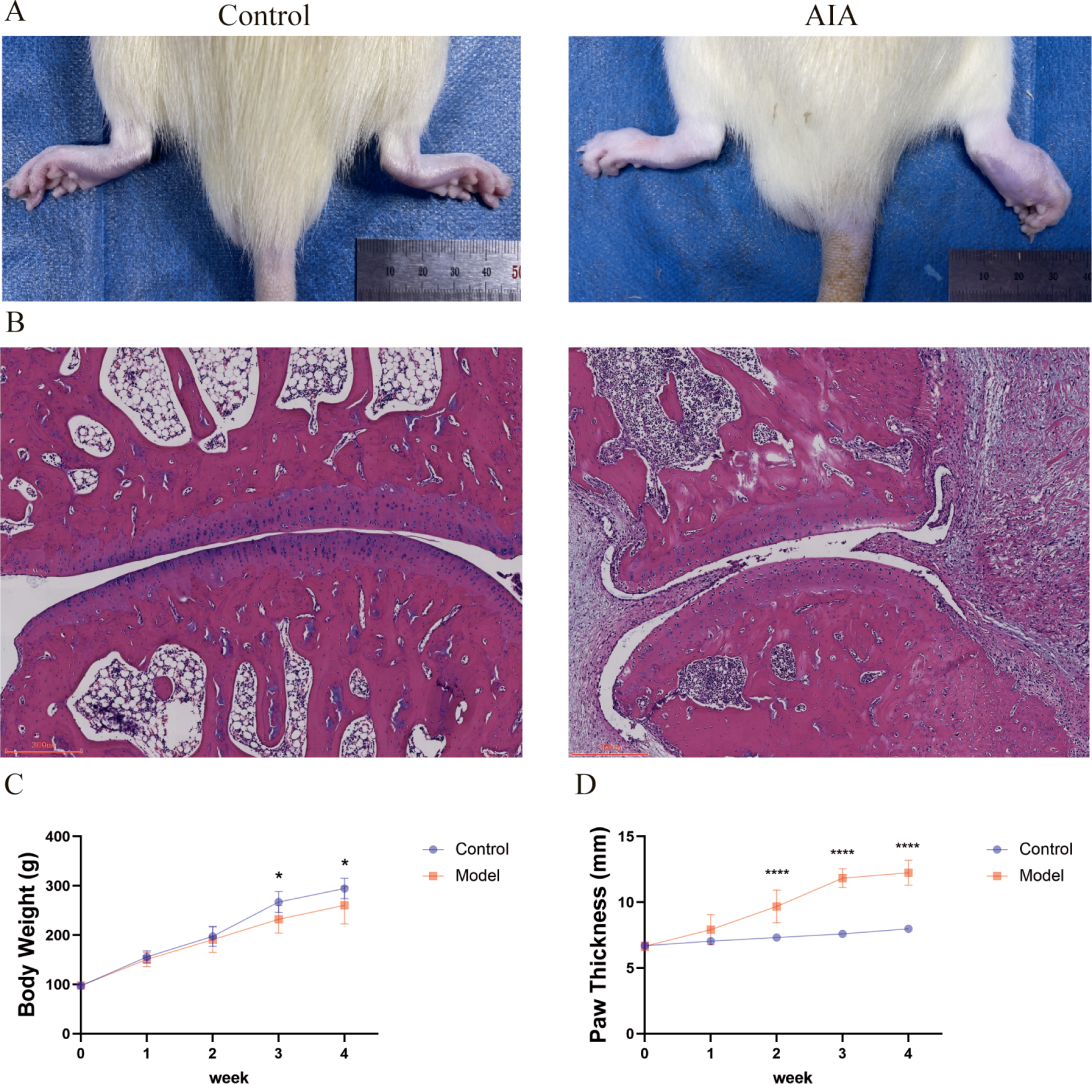
Paw swelling and pathological changes in normal rats and adjuvant-induced arthritis rats. Compared with normal rats, *P < 0.05, ****P < 0.0001. **(A)** Comparison of paw size between the control and AIA groups. **(B)** HE staining of rat joints. **(C)** Body weight changes in the control and AIA groups from 0 to 4 weeks. **(D)** Changes in paw thickness in control and CIA groups from 0 to 4 weeks.

Compared with rats in the normal group, AIA rats exhibited obvious redness, swelling, heat, and pain in the paws, with a significant increase in swelling (**Figure 9A**), indicating successful model establishment. Histopathological analysis of the paw tissue sections (H&E staining) demonstrated that AIA rats displayed significant synovial inflammatory hyperplasia and marked inflammatory cell infiltration (**Figure 9B**). All rats showed an increase in body weight; however, as compared with the control group, the AIA group had significantly lower body weight (*P* < 0.05) (**Figure 9C**) and significantly greater paw thickness (*P* < 0.0001) (**Figure 9D**).

### 3.7 Differences in expression levels of key PANoptosis proteins in paw synovial tissue between normal rats and adjuvant arthritis rats

**Figure 10:**
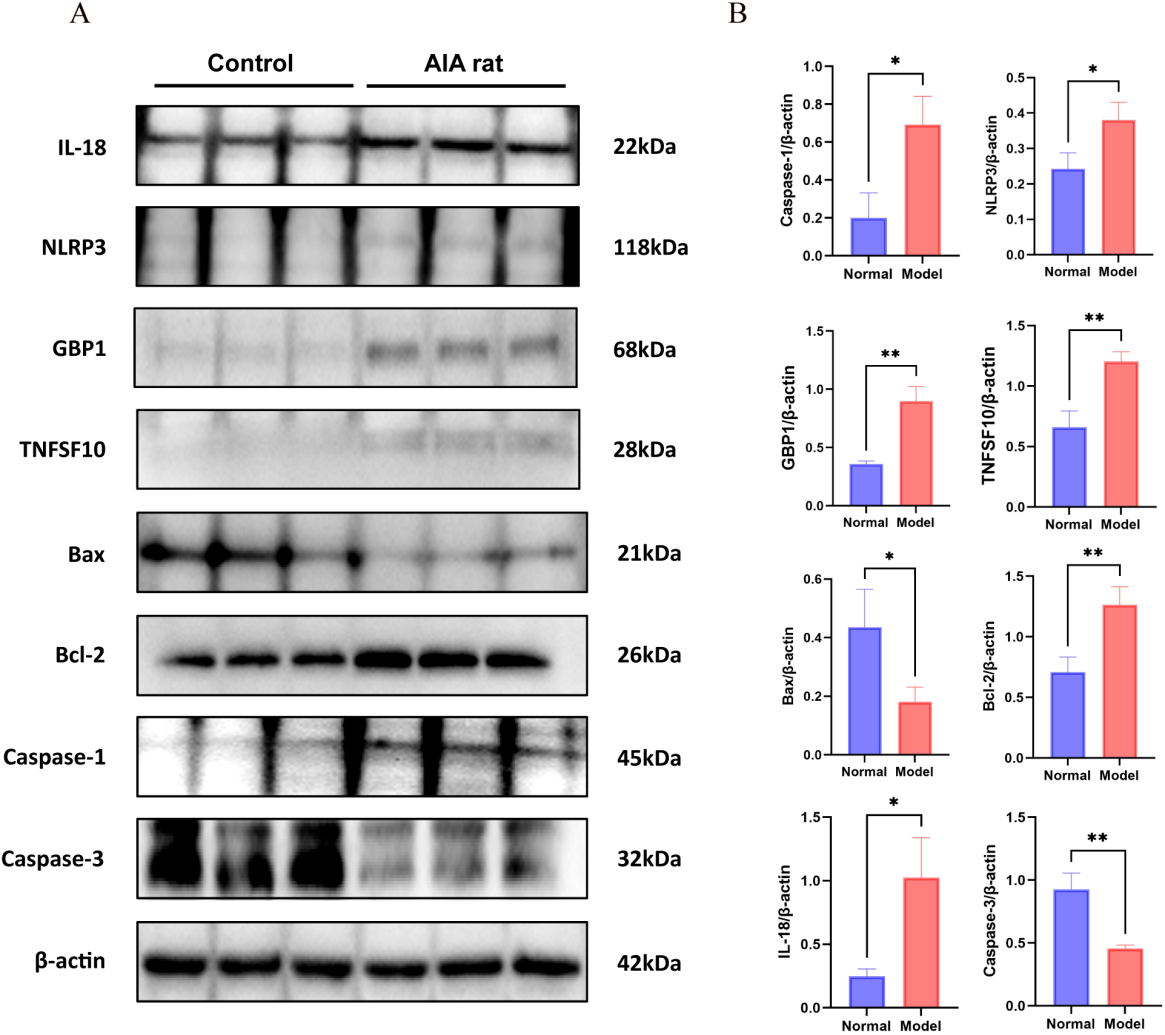
Differences in expression levels of key PANoptosis proteins in synovial tissue from normal rats and adjuvant arthritis rats. Compared with normal rats, *P < 0.05, **P < 0.01. **(A)** Western blot results. **(B)** Differential analysis between the normal and RA groups.

As shown in **Figure 10**, compared with rats in the normal group, the expression levels of key PANoptosis proteins including IL-18 (*P* < 0.05), NLRP3 (*P* < 0.05), GBP1 (*P* < 0.01), TNFSF10 (*P* < 0.01), Caspase-1 (*P* < 0.05) and Bcl-2 (*P* < 0.01) in the paw synovial tissue of rats in the model group were significantly increased, while the expression levels of Caspase-3 (*P* < 0.01) and Bax (*P* < 0.05) were significantly decreased. The results demonstrated that during the development of RA, the disease is closely associated with the PANoptosis of synovial cells in the joint synovial tissue.

## 4 Discussion

The core pathological feature of RA is chronic synovitis and invasive hyperplasia, which ultimately leads to joint damage (17). Focusing on the core pathological mechanism of RA, this study systematically explored the role of PANoptosis in RA following the logical chain of “bioinformatics analysis to predict core genes, immune infiltration analysis to interpret microenvironment background, *in vitro* cell experiments for verification, and *in vivo* animal experiments for confirmation. In the bioinformatics analysis phase, three RA synovial tissue datasets (GSE55235, GSE55457, and GSE55584) were integrated. After batch correction and differential expression analysis, 1293 RA-related differentially expressed genes were identified. Meanwhile, core genes involved in three cell death pathways (apoptosis, pyroptosis, and necroptosis) were mined from multiple databases, ultimately determining 85 key PANoptosis-related genes. By intersecting RA differentially expressed genes with key PANoptosis genes and combining with PPI network analysis, IL-18, NLRP3, GBP1, and TNFSF10 were identified as core targets. KEGG pathway enrichment analysis showed that these genes were significantly enriched in the NOD-like receptor signaling pathway and apoptosis-related signaling pathways, while GO functional enrichment indicated their involvement in biological processes such as pyroptosis and innate immune response, providing a clear direction for subsequent experiments.

**Figure 11:**
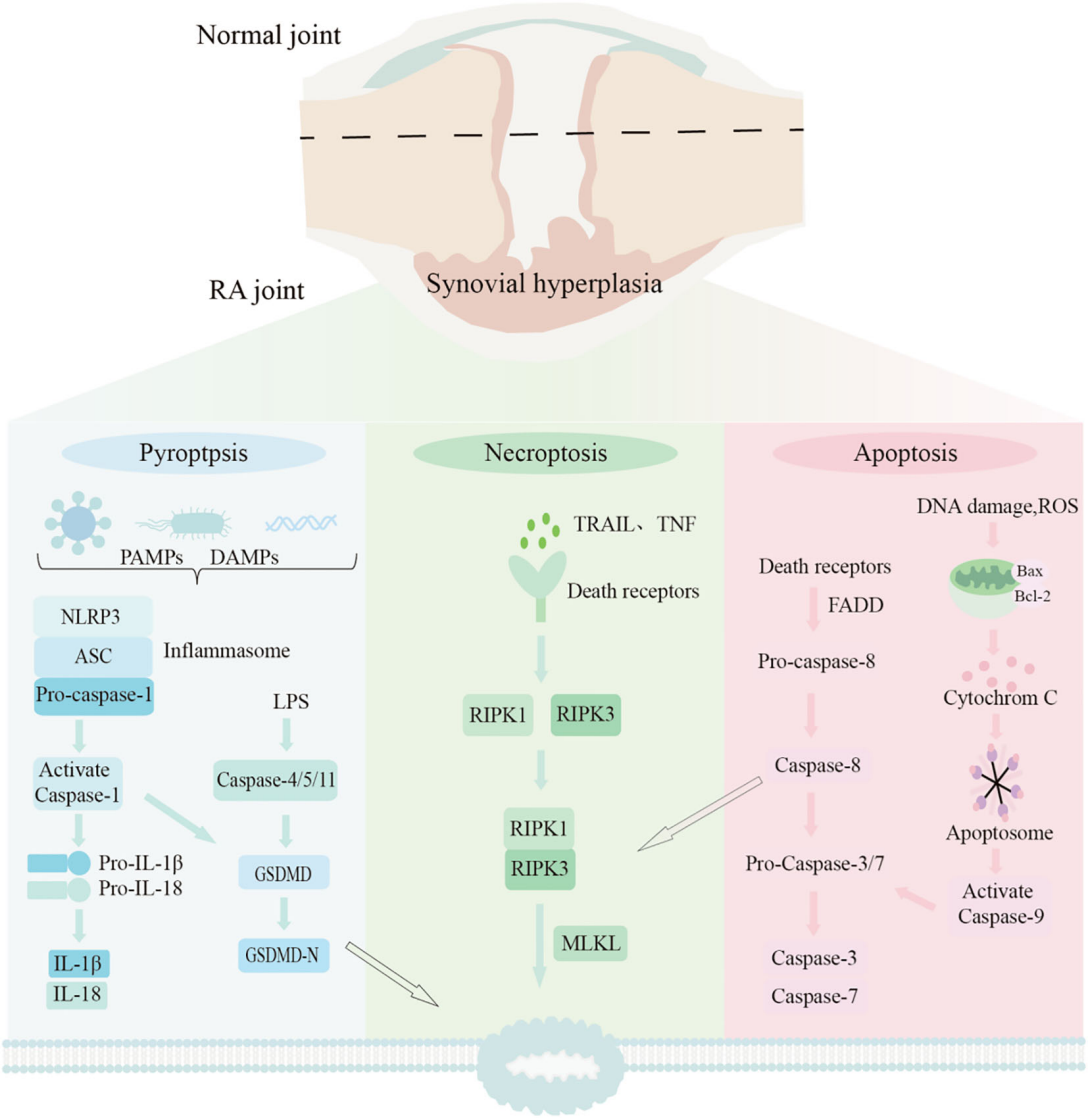
Mechanistic diagram of PANoptosis and RA synovial proliferation.

PANoptosis, as a newly elucidated inflammatory programmed cell death pathway integrating key features of pyroptosis, apoptosis, and necroptosis, is gradually revealing its molecular mechanisms, regulatory networks, and roles in disease, with significant advances particularly in tumor immunity and infection (**Figure 11**). However, its specific mechanisms and pathogenic significance in autoimmune diseases, particularly RA, remain understudied areas (18–20).On this basis, immune infiltration analysis using the CIBERSORT algorithm revealed the characteristic immune microenvironment of RA synovial tissue: proinflammatory/effector immune cell subsets (M0/M1 macrophages, effector T cells) were significantly enriched, whereas the normal control group was dominated by homeostatic cells such as regulatory T cells (Tregs) and activated mast cells. This microenvironment not only explains why PANoptosis is abnormally activated in RA: highly activated macrophages and T cells may be susceptible targets or executors of PANoptosis, but also clarifies the cellular targets of core genes, laying a foundation for the selection of subsequent experimental models (21).

*In vitro* cell experiments further verified the conclusions from bioinformatics prediction and immune infiltration. Using RAFLS as the research object, LPS induction was applied to simulate the *in vivo* proinflammatory microenvironment. Western Blot detection showed that compared with normal synovial fibroblasts (HFLS), the expression levels of IL-18, NLRP3, GBP1, TNFSF10, Caspase-1, and the anti-apoptotic protein Bcl-2 were significantly upregulated in RAFLS, while the expression levels of the apoptotic executor Caspase-3 and the pro-apoptotic protein Bax were significantly downregulated. This confirmed that PANoptosis in RAFLS exhibits an imbalanced state of “activated pyroptosis/necroptosis and inhibited apoptosis”. This imbalance not only leads to abnormal proliferation of RAFLS but also exacerbates synovial inflammation by releasing proinflammatory factors. To confirm this mechanism *in vivo*, an AIA rat model was established. Macroscopic phenotypes showed that AIA rats exhibited redness, swelling, heat, and pain in the paws, increased paw swelling and thickness, and slowed weight gain. H&E staining confirmed synovial inflammatory hyperplasia and cellular infiltration, indicating successful model establishment. WB detection further revealed that the expression changes of key PANoptosis proteins in the synovial tissue of AIA rats were highly consistent with those in cell experiments, demonstrating that PANoptosis imbalance is not an accompanying phenomenon in RA pathology but a key mechanism driving synovial inflammation and tissue destruction, thus completing the verification chain from *in vitro* to *in vivo*.

This study focused on RA, investigating the expression of key PANoptosis-related genes and proteins in RA synovial tissue and cells. This study contributes to a better understanding of PANoptosis as a potential core pathogenic mechanism in RA, and offers preliminary experimental evidence that supports its further investigation as a therapeutic target. Through bioinformatics analysis of PANoptosis-related genes in RA synovial tissue, four key differentially expressed genes—IL-18, NLRP3, GBP1, and TNFSF10—were identified. KEGG pathway enrichment analysis further revealed these PANoptosis genes, differentially expressed in human RA synovial tissue, were significantly enriched in the NOD-like receptor signaling pathway and apoptosis-related signaling pathways: Caspase-3, an apoptosis executor, acts downstream of the Bcl-2/Bax-regulated mitochondrial apoptosis pathway to directly mediate programmed cell death (22); Caspase-1, a key pyroptosis effector, can be activated by overactivated NLRP3 inflammasomes, leading to Gasdermin-D cleavage, membrane pore formation, cell lysis, and release of potent proinflammatory factors (e.g., IL-1β, IL-18) that exacerbate synovial inflammation and tissue destruction (23, 24).

Experimental validation demonstrates that the four key genes are closely associated with distinct branches of PANoptosis and highly correlated with the abnormal immune cell subsets identified via immune infiltration analysis. IL-18, a critical proinflammatory cytokine, is well documented to be involved in RA pathogenesis. It stimulates the secretion of IFN-γ, which in turn activates synovial macrophages, consistent with the enrichment of M0/M1 macrophages observed in the infiltration analysis, promotes the synthesis of proinflammatory cytokines such as TNF-α and IL-1β as well as various chemokines, and acts on articular cartilage and synovial tissue to induce inflammatory damage. Furthermore, IL-18 drives synovial fibroblasts to release vascular endothelial growth factor, thereby inducing neovascularization closely linked to intra-articular angiogenesis in RA patients (25). The inflammatory response driven by the NLRP3 inflammasome also contributes to RA pathogenesis by regulating diverse cell types, such as modulating Th17 cells, a subset of helper T cells, to secrete IL-17A and TNF-α, which mediate proinflammatory responses resulting in tissue destruction, articular cartilage impairment, and bone erosion (26). GBP1, an interferon-induced protein, is strongly linked to the imbalance of synovial macrophages, and its role in regulating inflammation points to its potential function in the PANoptosis network (27, 28). TNFSF10, TRAIL, classified as a necroptosis-related gene, promotes the proliferation of RA synovial fibroblasts, and its expression level is significantly associated with RA severity (29).

These four genes regulate different branches of PANoptosis, respectively, and synergistically drive the progression of RA. The innovative value of this study lies in confirming the role of PANoptosis in RA for the first time through a complete logical chain, which fills the research gap regarding the function of PANoptosis in the core pathogenesis of RA; the screened core genes can serve as novel biomarkers for the early diagnosis of RA; and it also provides experimental evidence and theoretical support for the development of RA therapeutic strategies targeting the PANoptosis pathway and intervening in the activity of dysregulated immune cells, which is of great significance for deepening the understanding of RA pathogenesis and promoting clinical translation.

Integrating the results of multidimensional studies, this research confirmed that IL-18, NLRP3, GBP1, and TNFSF10 collectively form the core nodes of the PANoptosis regulatory network in RA synovial tissue: IL-18 promotes macrophage activation and angiogenesis, NLRP3 activates pyroptosis via inflammasomes, GBP1 is associated with macrophage imbalance, and TNFSF10 promotes RAFLS proliferation. These four genes regulate different branches of PANoptosis and synergistically drive the progression of RA. The innovative value of this study lies in three aspects: first, it is the first to confirm the role of PANoptosis in RA through a complete logical chain, filling the research gap in the role of PANoptosis in the core pathogenesis of RA; second, the screened core genes can serve as novel biomarkers for the early diagnosis of RA; third, it provides experimental evidence and theoretical support for the development of RA therapeutic strategies targeting the PANoptosis pathway and intervening in the activity of dysregulated immune cells, which is of great significance for deepening the understanding of RA pathogenesis and promoting clinical translation.

Although this study systematically revealed the expression profiles and potential roles of PANoptosis-related molecules in synovial hyperplasia of rheumatoid arthritis through bioinformatics analysis, *in vitro* cell models, and *in vivo* animal experiments, certain limitations remain. First, experimental validation primarily relied on RAFLSs and AIA rat models. While these models serve as classical tools for studying RA pathogenesis and effectively mimic key features of inflammatory activation and synovial hyperplasia, they cannot fully replicate the complex cellular heterogeneity, chronic immune microenvironment, and individual genetic backgrounds present in human RA synovial tissue. Second, Western blot analysis can only reveal changes in protein expression, limiting its ability to confirm the effects of PANoptosis on driving the invasive or proliferative behavior of RAFLS. Given the aforementioned limitations, we plan to conduct gain-of-function and loss-of-function experiments in existing cellular and animal models through siRNA/shRNA-mediated knockdown of key molecules and PANoptosis pathway-specific inhibitors. This will clarify their direct regulatory roles in synovial cell activation, proliferation, and inflammatory phenotypes. Subsequent work will involve collecting synovial surgical specimens from clinical RA patients, with osteoarthritis patients serving as controls. Through protein and gene profiling of primary tissues, we will validate the correlations identified in this study between PANoptosis key molecules and RA disease activity, pathological features, and treatment response. This step serves as a critical bridge connecting mechanistic research to clinical translation, providing essential human histological evidence for evaluating the potential therapeutic value of targeting the PANoptosis pathway in RA.

## Data Availability

The datasets presented in this study can be found in online repositories. The names of the repository/repositories and accession number(s) can be found in the article/**Supplementary Material**.
