# Supplementary figures and images for: PANoptosis-related genes in rheumatoid arthritis synovial tissue: screening, validation, and functional implications
Source: Front Immunol. 2026 Jan 28;17:1737366. doi: 10.3389/fimmu.2026.1737366 (PMC12926709; doi:10.3389/fimmu.2026.1737366)

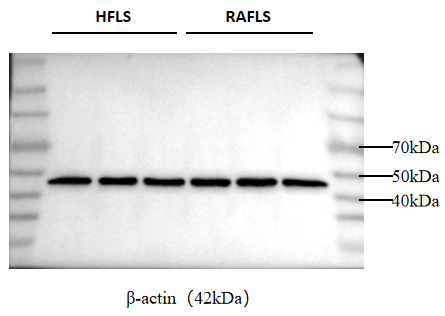


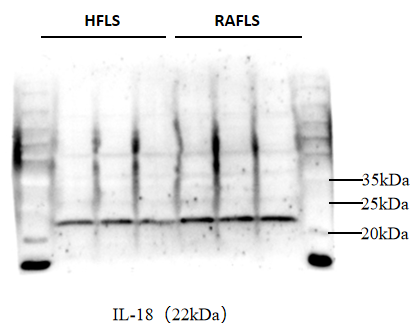

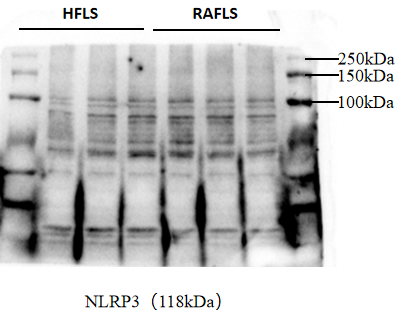

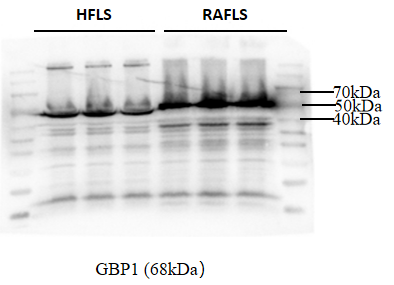

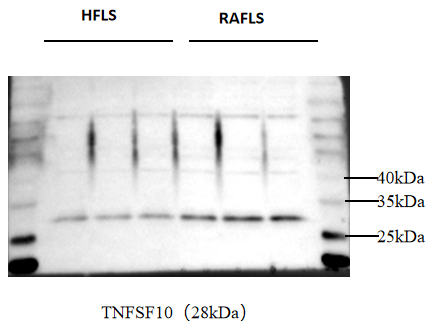

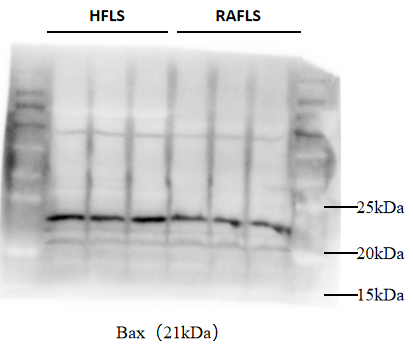

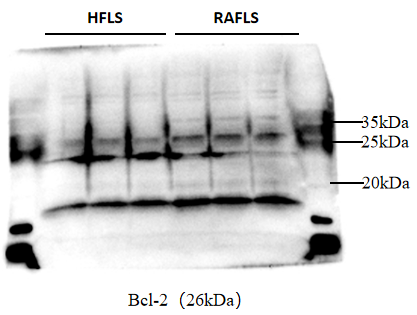

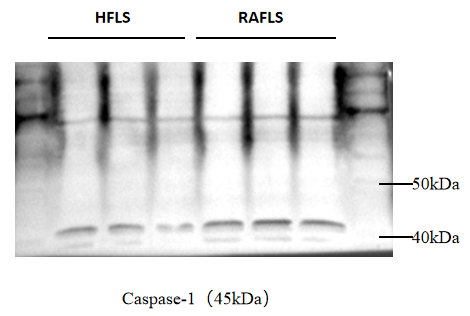

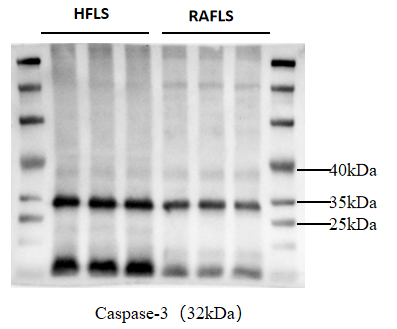


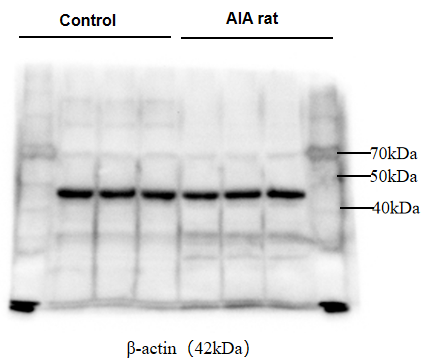

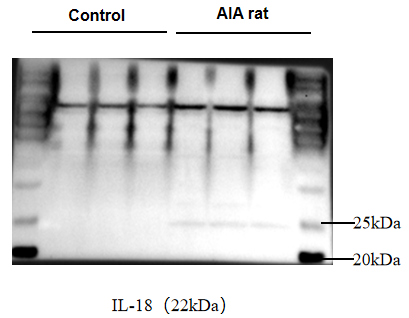


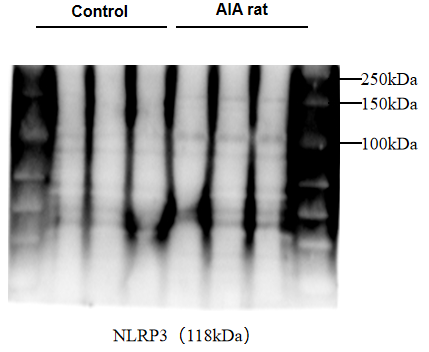

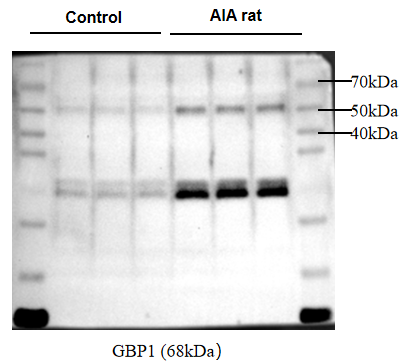

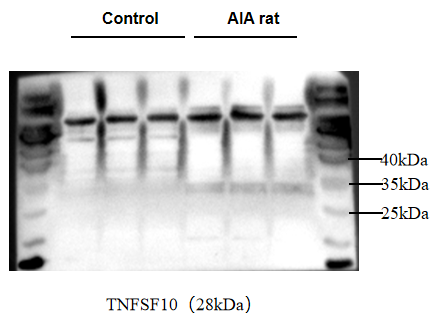

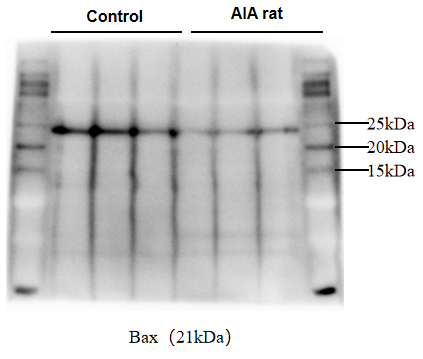

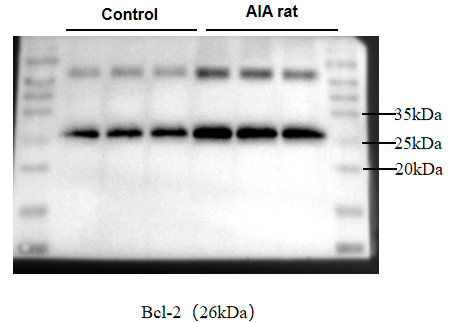

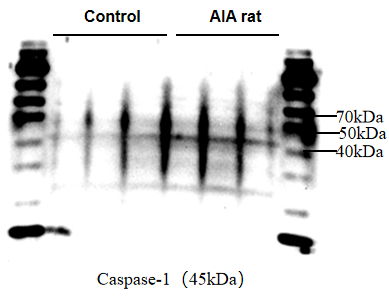


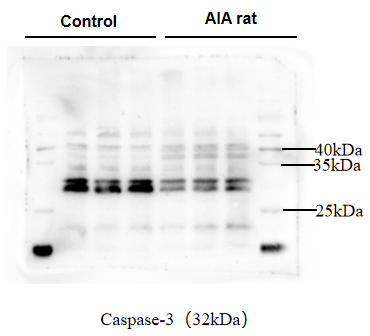

Supplement: Supplementary file 2 [file Table2.docx]
